# The Single-Case Reporting Guideline In BEhavioural Interventions (SCRIBE) 2016 Statement^†^

**DOI:** 10.1080/09602011.2016.1190533

**Published:** 2016-08-08

**Authors:** Robyn L. Tate, Michael Perdices, Ulrike Rosenkoetter, William Shadish, Sunita Vohra, David H. Barlow, Robert Horner, Alan Kazdin, Thomas Kratochwill, Skye McDonald, Margaret Sampson, Larissa Shamseer, Leanne Togher, Richard Albin, Catherine Backman, Jacinta Douglas, Jonathan J. Evans, David Gast, Rumen Manolov, Geoffrey Mitchell, Lyndsey Nickels, Jane Nikles, Tamara Ownsworth, Miranda Rose, Christopher H. Schmid, Barbara Wilson

**Affiliations:** ^a^ John Walsh Centre for Rehabilitation Research, The Kolling Institute of Medical Research, St Leonards, Australia; ^b^ Sydney Medical School Northern, The University of Sydney, Sydney, Australia; ^c^ Department of Neurology, Royal North Shore Hospital, St Leonards, Australia; ^d^ Discipline of Psychiatry, The University of Sydney, Sydney, Australia; ^e^ School of Social Sciences, Humanities and Arts, University of California, Merced, USA; ^f^ Department of Pediatrics, University of Alberta, Edmonton, Canada; ^g^ Center for Anxiety Related Disorders, Boston University, Boston, USA; ^h^ Department of Special Education and Clinical Services, University of Oregon, Eugene, USA; ^i^ Department of Psychology, Yale University, New Haven, USA; ^j^ School of Educational Psychology, University of Wisconsin—Madison, Madison, USA; ^k^ School of Psychology, University of New South Wales, Sydney, Australia; ^l^ Library and Media Services, Children’s Hospital of Eastern Ontario, Ottawa, Canada; ^m^ Clinical Epidemiology Program, Ottawa Hospital Research Institute, Ottawa, Canada; ^n^ School of Epidemiology, Public Health and Preventive Medicine, University of Ottawa, Ottawa, Canada; ^o^ Discipline of Speech Pathology, The University of Sydney, Sydney, Australia; ^p^ Department of Occupational Science and Occupational Therapy, University of British Columbia, Vancouver, Canada; ^q^ Department of Communication and Clinical Allied Health, La Trobe University, Melbourne, Australia; ^r^ Institute of Health and Wellbeing, University of Glasgow, Glasgow, UK; ^s^ Department of Special Education, University of Georgia, Athens, USA; ^t^ Department of Behavioural Sciences Methods, University of Barcelona, Barcelona, Spain; ^u^ Discipline of General Practice, University of Queensland, Brisbane, Australia; ^v^ Department of Cognitive Sciences, Macquarie University, Sydney, Australia; ^w^ Centre for Clinical Research, University of Queensland, Brisbane, Australia; ^x^ School of Applied Psychology, Griffith University, Brisbane, Australia; ^y^ School of Public Health, Brown University, Providence, USA; ^z^ Department of Neuropsychology, Oliver Zangwill Centre, Ely, UK

**Keywords:** Single-case design, methodology, reporting guidelines, publication standards

## Abstract

We developed a reporting guideline to provide authors with guidance about what should be reported when writing a paper for publication in a scientific journal using a particular type of research design: the single-case experimental design. This report describes the methods used to develop the Single-Case Reporting guideline In BEhavioural interventions (SCRIBE) 2016. As a result of 2 online surveys and a 2-day meeting of experts, the SCRIBE 2016 checklist was developed, which is a set of 26 items that authors need to address when writing about single-case research. This article complements the more detailed SCRIBE 2016 Explanation and Elaboration article (Tate et al., 2016) that provides a rationale for each of the items and examples of adequate reporting from the literature. Both these resources will assist authors to prepare reports of single-case research with clarity, completeness, accuracy, and transparency. They will also provide journal reviewers and editors with a practical checklist against which such reports may be critically evaluated. We recommend that the SCRIBE 2016 is used by authors preparing manuscripts describing single-case research for publication, as well as journal reviewers and editors who are evaluating such manuscripts.

**SCIENTIFIC ABSTRACT**

Reporting guidelines, such as the Consolidated Standards of Reporting Trials (CONSORT) Statement, improve the reporting of research in the medical literature (Turner et al., 2012). Many such guidelines exist and the CONSORT Extension to Nonpharmacological Trials (Boutron et al., 2008) provides suitable guidance for reporting between-groups intervention studies in the behavioural sciences. The CONSORT Extension for N-of-1 Trials (CENT 2015) was developed for multiple crossover trials with single individuals in the medical sciences (Shamseer et al., 2015; Vohra et al., 2015), but there is no reporting guideline in the CONSORT tradition for single-case research used in the behavioural sciences. We developed the Single-Case Reporting guideline In BEhavioural interventions (SCRIBE) 2016 to meet this need. This Statement article describes the methodology of the development of the SCRIBE 2016, along with the outcome of 2 Delphi surveys and a consensus meeting of experts. We present the resulting 26-item SCRIBE 2016 checklist. The article complements the more detailed SCRIBE 2016 Explanation and Elaboration article (Tate et al., 2016) that provides a rationale for each of the items and examples of adequate reporting from the literature. Both these resources will assist authors to prepare reports of single-case research with clarity, completeness, accuracy, and transparency. They will also provide journal reviewers and editors with a practical checklist against which such reports may be critically evaluated.

University courses generally prepare students of the behavioural sciences very well for research using parallel, between-groups designs. By contrast, single-case methodology is “rarely taught in undergraduate, graduate and postdoctoral training” (Kazdin, 2011, p. vii). Consequently, there is a risk that researchers conducting and publishing studies using single-case experimental designs (and journal reviewers of such studies) are not necessarily knowledgeable about single-case methodology nor well trained in using such designs in applied settings. This circumstance, in turn, impacts the conduct and report of single-case research. Even though single-case experimental intervention research has comparable frequency to between-groups research in the aphasiology, education, psychology, and neurorehabilitation literature (Beeson & Robey, 2006; Perdices & Tate, 2009; Shadish & Sullivan, 2011), evidence of inadequate and incomplete reporting is documented in multiple surveys of this literature in different populations (Barker, Mellalieu, McCarthy, Jones, & Moran, 2013; Didden, Korzilius, van Oorsouw, & Sturmey, 2006; Maggin, Chafouleas, Goddard, & Johnson, 2011; Smith, 2012; Tate, Perdices, McDonald, Togher, & Rosenkoetter, 2014).

To address these issues we developed a reporting guideline, entitled the Single-Case Reporting guideline In BEhavioural interventions (SCRIBE) 2016, to assist authors, journal reviewers and editors to improve the reporting of single-case research. This Statement provides the methodology and development of the SCRIBE 2016. The companion SCRIBE 2016 Explanation and Elaboration (E&E) article (Tate et al., 2016) provides detailed background to and rationale for each of the 26 items in the SCRIBE checklist, along with examples of adequate reporting in the published literature.

The SCRIBE 2016 Statement is intended for use with the family of single-case experimental designs** Single-case methodology is defined as the intensive and prospective study of the individual in which (a) the intervention/s is manipulated in an experimentally controlled manner across a series of discrete phases, and (b) measurement of the behavior targeted by the intervention is made repeatedly (and, ideally, frequently) throughout all phases. Professional guidelines call for the experimental effect to be demonstrated on at least three occasions by systematically manipulating the independent variable (Horner et al., 2005; Kratochwill et al., 2010, 2013). This criterion helps control for the confounding effect of extraneous variables that may adversely affect internal validity (e.g., history, maturation) and allows a functional cause and effect relationship to be established between the independent and dependent variables. used in the behavioural sciences. It applies to four prototypical designs (withdrawal/reversal, multiple baseline, alternating-treatments, and changing-criterion designs), including combinations and variants of these designs, as well as adaptive designs. Figure 1 presents the common designs using a single case based on surveys in the literature (see, e.g., Perdices & Tate, 2009; Shadish & Sullivan, 2011).

**Figure 1.**
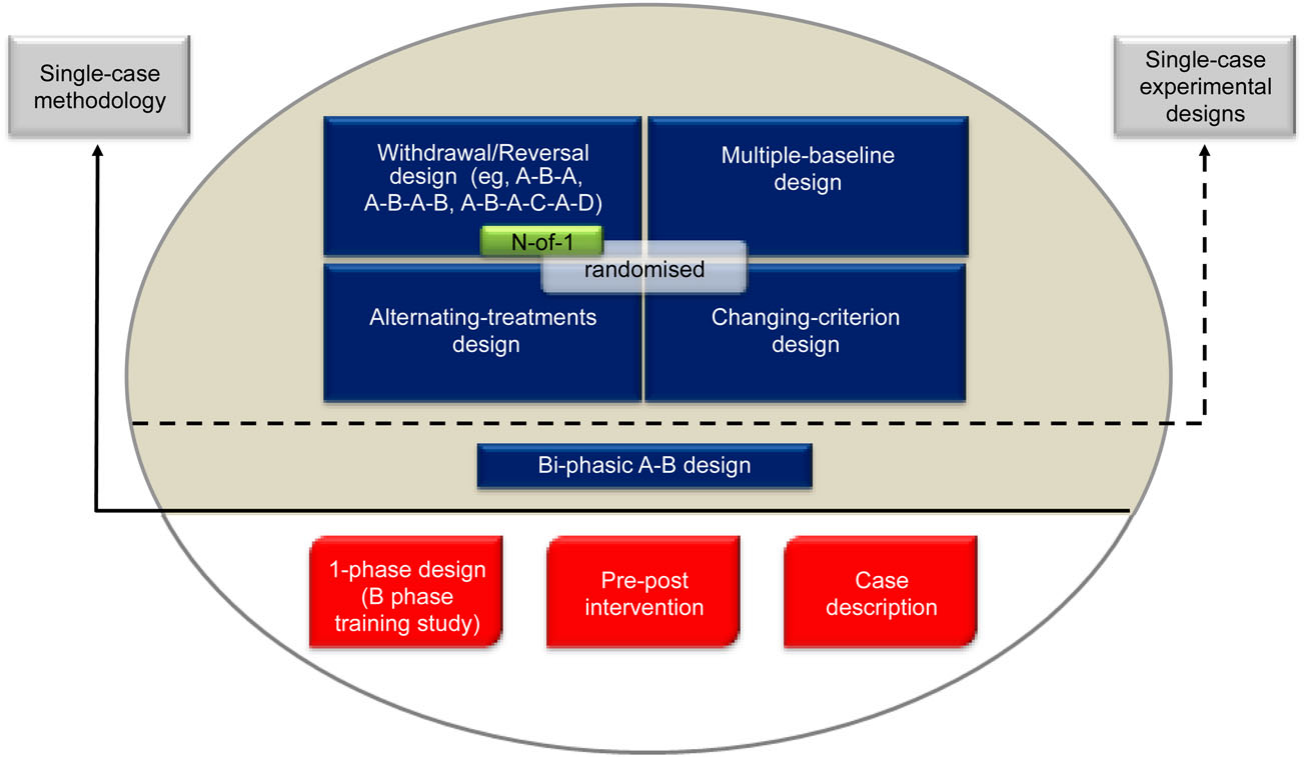
Common designs in the literature using a single participant. Reproduced from the expanded manual for the Risk of Bias in N-of-1 Trials (RoBiNT) Scale (Tate et al., 2015) with permission of the authors; an earlier version of the figure, taken from the original RoBiNT Scale manual (Tate et al., 2013a) was also published in 2013 (Tate et al., 2013b).

The figure mainly draws on the behavioural sciences literature, which includes a broad range of designs using a single participant. Only those designs above the solid horizontal line use single-case methodology (i.e., an intervention is systematically manipulated across multiple phases during each of which the dependent variable is measured repeatedly and, ideally, frequently). None of the designs below the solid horizontal line meets these criteria and they are not considered single-case experiments: The B-phase training study comprises only a single (intervention) phase; the so-called “pre–post” study does not take repeated measurements *during* the intervention phase; and the case description is a report, usually compiled retrospectively, that is purely descriptive without systematic manipulation of an intervention.

The A-B design, also labelled “phase change without reversal” (Shadish & Sullivan, 2011), is widely regarded as the basic single-case design. It differs from the “pre–post” study in that measurement of the dependent variable occurs *during* the intervention (B) phase. In the Figure, we place the A-B design in an intermediate position between the nonexperimental single-case designs (below the solid horizontal line) and the four experimental designs above the dotted horizontal line because it has weak internal validity, there being no control for history or maturation, among other variables. As a result, it is regarded as a quasiexperimental design (Barlow, Nock, & Hersen, 2009).

Designs above the dotted horizontal line are experimental in that the control of threats to internal validity is stronger than in the A-B design. Nonetheless, within each class of design the adequacy of such controls and whether or not the degree of experimental control meets design standards (see Horner et al., 2005; Kratochwill et al., 2013) vary considerably (cf. A-B-A vs. A-B-A-B; multiple-baseline designs with two vs. three baselines/tiers). Consequently, reports of these designs in the literature have variable scientific quality and features of internal and external validity can be evaluated with scales measuring scientific robustness in single-case designs, such as described in Maggin, Briesch, Chafouleas, Ferguson, and Clark (2014) and Tate et al. (2013b).

The structure of the four prototypical experimental designs in Figure 1 differ significantly: The withdrawal/reversal design systematically applies and withdraws an intervention in a sequential manner, the multiple-baseline design systematically applies an intervention in a sequential manner that also has a staggered introduction across a particular parameter (e.g., participants, behaviours), the alternating/simultaneous-treatments design compares multiple interventions in a concurrent manner by rapidly alternating the application of the interventions, and the changing-criterion design establishes a number of hierarchically based criterion levels that are implemented in a sequential manner. Each of the single-case experimental designs has the capacity to introduce randomisation into the design (cf. the small grey rectangle within each of the designs in Figure 1), although in practice randomisation in single-case research is not common.

The medical *N*-of-1 trial is depicted within the withdrawal/reversal paradigm of Figure 1. The analogous reporting guide for the medical sciences, CONSORT Extension for *N*-of-1 Trials (CENT 2015; Shamseer et al., 2015; Vohra et al., 2015), is available for the reporting of medical *N*-of-1 trials. These trials consist of multiple cross-overs (described as challenge-withdrawal-challenge-withdrawal in Vohra *et al*.) in a single participant who serves as his or her own control, often incorporating randomisation and blinding.

As with other reporting guidelines in the CONSORT tradition, the SCRIBE 2016 does not make recommendations about how to design, conduct or analyse data from single-case experiments. Rather, its primary purpose is to provide authors with a checklist of items that a consensus from experts identified as the minimum standard for facilitating comprehensive and transparent reporting. This checklist includes the specific aspects of the methodology to be reported and suggestions about how to report. Consequently, readers are provided with a clear, complete, accurate, and transparent account of the context, plan, implementation and outcomes of a study. Readers will then be in a position to critically evaluate the adequacy of the study, as well as to replicate and validate the research. Clinicians and researchers who want guidance on how to design, conduct and analyse data for single-case experiments should consult any of the many current textbooks and reports (e.g., Barker, McCarthy, Jones, & Moran, 2011; Barlow et al., 2009; Gast & Ledford, 2014; Horner et al., 2005; Kazdin, 2011; Kennedy, 2005; Kratochwill et al., 2013; Kratochwill & Levin, 2014; Morgan & Morgan, 2009; Riley-Tillman & Burns, 2009; Vannest, Davis, & Parker, 2013), as well as recent special issues of journals (e.g., *Journal of Behavioral Education* in 2012, *Remedial and Special Education* in 2013, the *Journal of School Psychology* and *Neuropsychological Rehabilitation* in 2014, *Aphasiology* in 2015) and methodological quality recommendations (Horner et al., 2005; Kratochwill et al., 2013; Maggin et al., 2014; Smith, 2012; Tate et al., 2013b).

## Initial steps

The impetus to develop the SCRIBE 2016 arose during the course of discussion at the CENT consensus meeting in May 2009 in Alberta, Canada (see Shamseer et al., 2015; Vohra et al., 2015). The CENT initiative was devoted to developing a reporting guideline for a specific design and a specific discipline: *N*-of-1 trials in the medical sciences. At that meeting the need was identified for development of a separate reporting guideline for the broader family of single-case experimental designs as used in the behavioural sciences (see Figure 1).

A 13-member steering committee for the SCRIBE project was formed comprising a Sydney, Australia, executive (authors RLT, convenor, and SM, MP, LT, with UR appointed as project manager). An additional three members who had spearheaded the CENT initiative (CENT convenor, SV, along with MS and LS) were invited because of their experience and expertise in developing a CONSORT-type reporting guideline in a closely related field (*N*-of-1 trials). In order to ensure representation from experts in areas of single-case investigations in clinical psychology, special education and single-case methodology and data analysis, another five experts were invited to the steering committee (authors DHB, RH, AK, TK, and WS). Of course, other content experts exist who would have been eligible for the steering committee, but a guiding consideration was to keep the number of members to a reasonable size so that the project was manageable. In the early stages of the project, steering committee members were instrumental in item development and refinement for the Delphi survey.

The methodology used to develop the SCRIBE 2016 followed the procedures outlined by Moher, Schulz, Simera, and Altman (2010). At the time of project commencement, the literature on evidence of bias in reporting single-case research was very limited and it has only recently started to emerge. Members of the steering committee, however, were already knowledgeable about the quality of the existing single-case literature, which had prompted independent work in the United States (specifically in compiling competency standards of design and evidence; Hitchcock et al., 2014; Horner et al., 2005; Kratochwill et al., 2010, 2013) and Australia (in developing an instrument to evaluate the scientific quality of single-case experiments; Tate et al., 2008, 2013b). No reporting guideline, in the CONSORT tradition, emerged from literature review.

Since commencement of the SCRIBE project, a reporting guide for single-case experimental designs was published by Wolery, Dunlap, and Ledford (2011). That guide was not developed following the same series of steps as in previously developed reporting guidelines such as those of the CONSORT family (see Moher et al., 2011) and is not as comprehensive as the CONSORT-type guidelines on which the current project is based, covering about half of the items in the SCRIBE 2016. Nevertheless, the convergence between the recommendations of Wolery and colleagues regarding the need to report on features such as inclusion and exclusion criteria for participants, design rationale, operational definitions of the target behaviour versus the corresponding items presented in the SCRIBE 2016 is noteworthy and adds validity to the SCRIBE 2016. Funding for the SCRIBE project was obtained from the Lifetime Care and Support Authority of New South Wales, Australia. The funds were used to employ the project manager, set up and develop a web-based survey, hold a consensus meeting, and sponsor participants to attend the consensus meeting.

## Premeeting activities

### Methodology of the Delphi process

The Delphi technique is a group decision-making tool and consensus procedure that is well suited to establishing expert consensus on a given set of items (Brewer, 2007). The nature of the process allows for it to be conducted online, and responses can be given anonymously. The Delphi procedure consists of several steps, beginning with the identification, selection, and invitation of a panel of experts in the pertinent field to participate in the consensus process. Subsequently, the items are distributed to experts who rate the importance of each topic contained in the items. As we did for the present project, a Likert scale is often used, ranging from 1 to 10, whereby 1 indicates *very low importance* and 10 *very high importance*. All expert feedback is then collated and reported back to the panel, including the mean, standard deviation, and median for each item, a graph indicating the distribution of responses, as well as any comments made by other experts to inform further decision-making. When high consensus is achieved, which may take several rounds, the Delphi exercise is completed. Von der Gracht (2012) reviews a number of methods to determine consensus for the Delphi procedure. Methods include using the interquartile range (IQR), with consensus operationalised as no more than 2 units on a 10-unit scale.

### The SCRIBE Delphi procedure

A set of potential items was drawn up by the SCRIBE steering committee for the Delphi survey. The items initially came from two sources available at the time: (a) those identified in a systematic review previously conducted by the CENT group (Punja et al., in press), and subsequently refined during the CENT consensus meeting process, and (b) items used to develop the Single-Case Experimental Design Scale published by the Sydney-based members as part of an independent project (Tate et al., 2008). Steering committee members suggested additional items, as well as rephrasing of existing items. We formatted the resulting 44 initial items for distribution in the Delphi exercise, using an online survey tool, SurveyMonkey.

**Figure 2.**
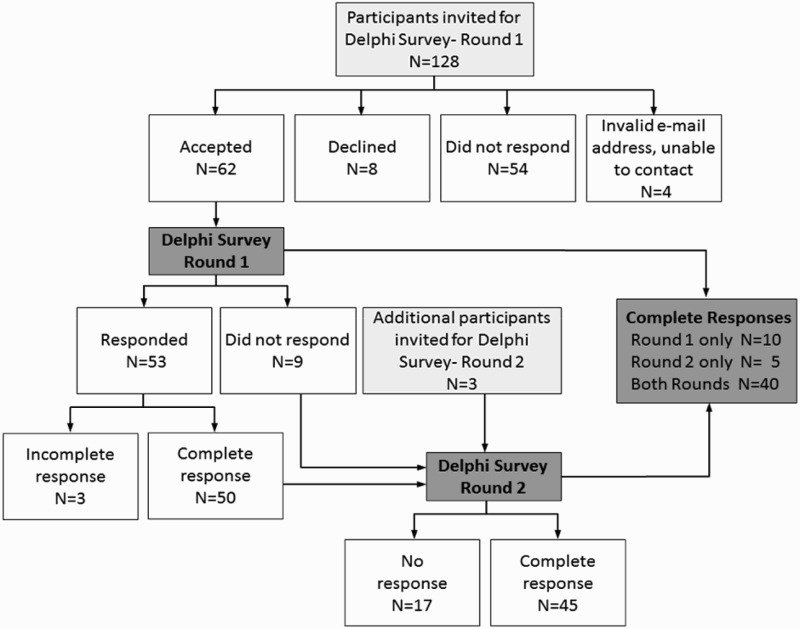
Flow diagram of the Delphi surveys.

Two rounds of a Delphi survey were conducted in April and September 2011. Figure 2 provides a flow diagram of the Delphi survey participants. In total, we identified 131 experts worldwide as potential Delphi panel members (128 for the initial round and an additional three participants were added at Round 2) based on their track record of published work in the field of single-case research (either methodologically or empirically based) and/or reporting guideline development. We used several strategies to identify suitable respondents. The Sydney executive drew up lists of authors who published single-case experimental designs in the behavioural sciences, by consulting reference lists of books and journal articles and our PsycBITE database (www.psycbite.com). We examined the quality of authors’ work, as described in their reports, using our methodological quality scale (Tate et al., 2008), and invited authors of scientifically sound reports. In addition, we conducted Google searches of editorial board members of journals that were known to publish single-case reports, as well as the authors publishing in such journals and evaluated the quality of their work. Finally, steering committee members made recommendations of suitable authors. This group of 131 invitees represents a sample of all world experts. We distributed invitations by e-mail for ease of communication and speed of contact. An “opt-in” consent arrangement was used and thus consent to participate required the invitee’s active response. Of the pool of 128 invitations for Round 1, 54 did not respond to the invitation (we sent one reminder e-mail), eight did respond but declined (mainly on the grounds of not having sufficient time), and four e-mail addresses were undeliverable. The remaining 62 responders who consented to participate in Round 1 were sent the survey link.

In Round 1, 53 of 62 consenting experts responded within the 2-week time frame of the survey, with 50 providing a complete data set of responses to the original set of 44 items. Results were entered into a database. Importance ratings of the items were uniformly high, with no item receiving a group median rating <7/10. The items thus remained unrevised for Round 2, which was conducted to elicit additional comment on the items. These decision-making criteria are compatible with that used in the development of the CENT 2015, which excluded items with mean importance ratings <5/10 (Vohra et al., 2015).

For Round 2, the survey link was sent to 59 of the original 62 consenting participants to Round 1 (the three participants who consented but did not complete Round 1 did not provide reasons for their early discontinuance and were not recontacted), and an additional three experts recommended by steering committee members. Graphed results were provided to respondents, along with anonymous comments on the items from the other panel members. A complete data set of responses for Round 2 was collected from 45 participants. Again, the ratings of importance for each item were mostly very high, all items having median importance ratings of at least 8/10, but the range of responses decreased. According to the criteria of Von der Gracht (2012) consensus was achieved for 82% of items (36/44) which had IQRs of 2 or less on the 10-point scale. The remaining eight items had IQRs from 2.25 to 4 and were discussed in detail at the consensus meeting.

As depicted in Figure 2, across the two rounds of the Delphi exercise 65/131 invited experts consented to participate (62 participants in Round 1 and an additional three participants in Round 2). Forty participants provided a complete data set of responses to both Round 1 and Round 2, representing a 62% response rate (40/65). The 40 responders represented 31% of the total of 131 experts invited to participate in the survey.

## Consensus meeting

Sixteen world experts in single-case methodology and reporting guideline development attended a 2-day consensus meeting, along with the Sydney executive and two research staff. Representation included clinical-research content experts in clinical and neuropsychology, educational psychology and special education, medicine, occupational therapy, and speech pathology; as well as single-case methodologists and statisticians; journal editors and a medical librarian; and guideline developers. Delegates met in Sydney on 8 and 9 December 2011. Each participant received a folder which contained reference material pertinent to the SCRIBE project, and results from both rounds of the Delphi survey. Each of the Delphi items contained a graph of the distribution of scores, the mean and median scores of each round of the survey, along with the delegate’s own scores when s/he completed the Delphi surveys.

The meeting commenced with a series of brief presentations from steering committee members on the topics of reporting guideline development, single-case methods and terminology, evolution of the SCRIBE project, and description of the CENT. Results of the Delphi survey were then presented. Delegates had their folder of materials to consult and a PowerPoint presentation that projected onto a screen to facilitate discussion. A primary aim of the consensus meeting was to develop the final set of items for the SCRIBE checklist. The final stages of the meeting discussed the documents to be published, authorship, and knowledge dissemination strategy.

During the meeting the 44 Delphi items were discussed, item by item, over the course of four sessions, each led by two facilitators. The guiding principles for discussion were twofold. First, item content was scrutinised to ensure that (a) it captured the essence of the intended issue under consideration and (b) the scope of the item covered the necessary and sufficient information to be reported. Second, the relevance of the item was examined in terms of its capacity to ensure clarity and accuracy of reporting.

Three delegates at the consensus meeting (authors RLT and SM, and a research staff member, DW) took notes about the amalgamation and merging of items where applicable and refinements to wording of items. Final wording of items was typed, live-time, into a computer that projected onto a screen so that delegates could see the changes, engage in further discussion, give approval, and commit to the group decision. In addition, the meeting was audiotaped for the purpose of later transcription to have a record of the discussion of the items and inform the direction and points to describe in the E&E document.

**Figure 3.**
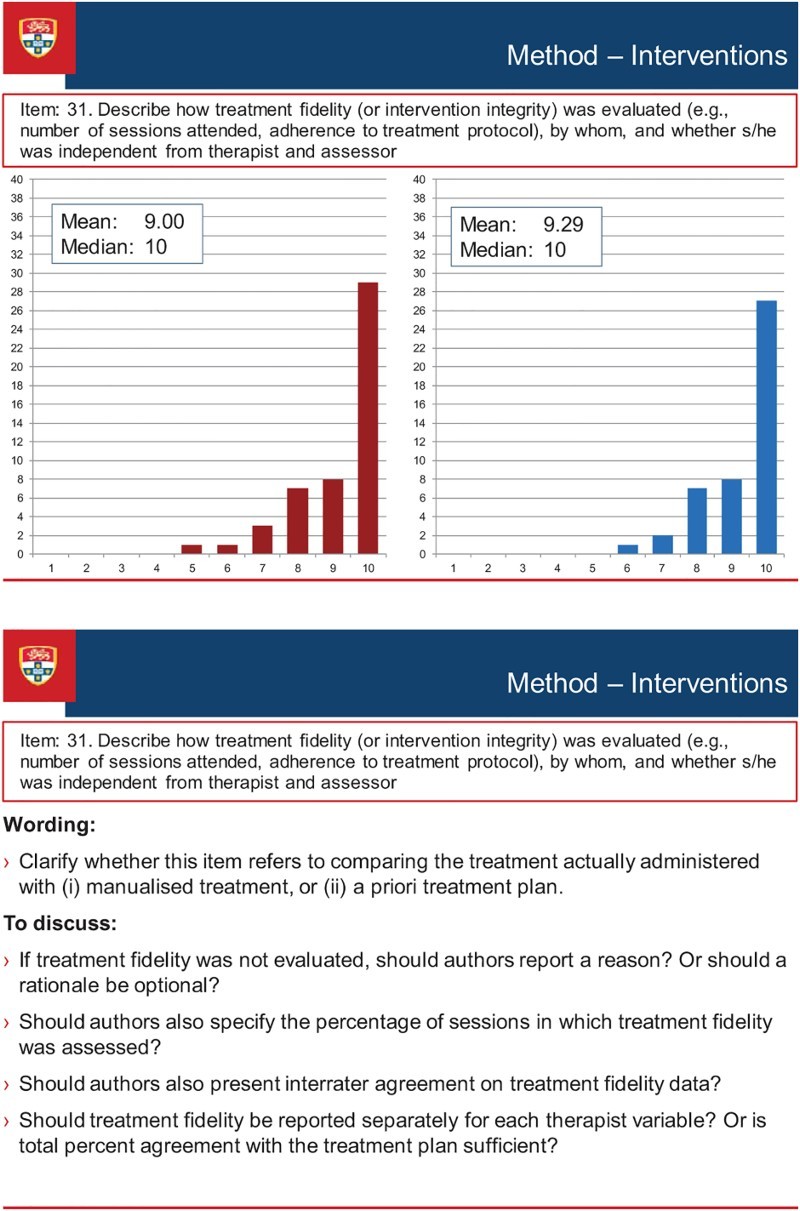
Screen-shot of a discussion item at the consensus meeting.

Figure 3 illustrates the discussion process that occurred during the consensus meeting. The figure presents a screen-shot of the PowerPoint presentation of one of the items (Item 31 of the Delphi survey, Treatment Fidelity, which was broadened to encompass procedural fidelity as a result of discussion at the consensus meeting, and became item 17 of the SCRIBE). The figure shows the results of each round of the Delphi survey (the results for Round 1 and Round 2 appear in the Figure as the left- and right-sided graphs respectively), along with discussion points. These points comprised comments made by the Delphi survey participants when completing the online surveys, as well as suggestions prepared by the Sydney executive that emerged from the consolidated comments. The points were used to stimulate discussion among the conference delegates, but discussion was not restricted to the prepared points.

By the end of the meeting, delegates reached consensus on endorsing 26 items that thus constitute the minimum set of reporting items comprising the SCRIBE 2016 checklist. The SCRIBE 2016 checklist consists of six sections in which the 26 aspects of report writing pertinent to single-case methodology are addressed. The first two sections focus on the title/abstract and introduction, each section containing two items. Section 3, method, consists of 14 items addressing various aspects of study methodology and procedure. Items include description of the design (e.g., randomisation, blinding, planned replication), participants, setting, ethics approval, measures and materials (including the types of measures, their frequency of measurement, and demonstration of their reliability), interventions, and proposed analyses. The results (Section 4) and discussion (Section 5), each contains three items. Section 6 (documentation) contains two items pertaining to protocol availability and funding for the investigation.

In total, 24 Delphi were merged into seven SCRIBE items because they referred to the same topics: (a) SCRIBE Item 5 (design) contained three Delphi items (design structure, number of sequences, and decision rules for phase change); (b) Item 8 (randomisation), two Delphi items (sequence and onset of randomisation); (c) Item 11 (participant characteristics), two Delphi items (demographics and aetiology); (d) Item 13 (approvals), two Delphi items (ethics approval and participant consent); (e) Item 14 (measures), nine Delphi items (operational definitions of the target behaviour, who selected it, how it was measured, independent assessor blind to phase, interrater agreement, follow-up measures, measures of generalisation and social validity, and methods to enhance quality of measurement); (f) Item 19 (results), two Delphi items (sequence completed and early stopping); and (g) Item 20 (raw data), four Delphi items (results, raw data record, access to raw data, and stability of baseline). One of the Delphi items relating to meta-analysis, was considered not to represent a minimum standard of reporting for single-case experimental designs and accordingly was deleted.

## Postmeeting activities

The audio recording of the 2-day consensus meeting was transcribed. The final guideline items were confirmed after close examination of the conference transcript and the SCRIBE 2016 checklist was developed (see Table 1). The meeting report was prepared and distributed to the steering committee members in June 2012. The Sydney executive then began the process of drafting background information sections for each item and integrating these with the broader literature for the E&E article. Multiple versions of the E&E article were distributed over the next 2 years to the steering committee members for their comment and subsequent versions incorporated the feedback.

**Table 1.** The Single-Case Reporting Guideline in BEhavioural Interventions (SCRIBE) 2016 checklist.

Item number	Topic	Item description
TITLE and ABSTRACT		
1	Title	Identify the research as a single-case experimental design in the title
2	Abstract	Summarize the research question, populalion, design, methods including in!ervention/s (independent variable/s) and target behavior/s and any other outcome/s (dependent variable/s), results, and conclusions
INTRODUCTION		
3	Scientific background	Describe the scientific background to identify issue/s under analysis, current scientific knowledge, and gaps in that knowledge base
4	Aims	State the purpose/aims of the study, research question/s, and, if applicable, hypotheses
METHOD		
	DESIGN	
5	Design	ldemify the design (e.g., withdrawal/reversal, multiple-baseline, alternating-treatments, changing-criterion, some combination thereof, or adaptive design) and describe the phases and phase sequence (whether determined *a priori* or data-driven) and, if applicable, criteria for phase change
6	Procedural changes	Describe any procedural changes that occurred during the course of the investigation after the start of the study
7	Replication	Describe any planned replication
8	Randomization	State whether randomization was used, and if so, describe the randomization method and the elements of the study that were randomized
9	Blinding	State whether blinding/masking was used, and if so, describe who was blinded/masked
	PARTICIPANT/S or UNIT/S	
10	Selection criteria	State the inclusion and exclusion criteria, if applicable, and the method of recruitment
11	Participant characteristics	For each participant, describe the demographic characteristics and clinical (or other) features relevant to the research question, such that anonymity is ensured
	CONTEXT	
12	Setting	Describe characteristics of the setting and location where the study was conducted
	APPROVALS	
13	Ethics	State whether ethics approval was obtained and indicate if and how informed consent and/or assent were obtained
	MEASURES and MATERIALS	
14	Measures	Operationally define all target behaviors and outcome measures, describe reliability and validity, state how they were selected, and how and when they were measured
15	Equipment	Clearly describe any equipment and/or materials (e.g., technological aids, biofeedback, computer programs, intervention manuals or other material resources) used to measure target behavior/s and other outcome/s or deliver the interventions
	INTERVENTIONS	
16	Intervention	Describe the intervention and control condition in each phase, including how and when they were actually administered, with as much detail as possible to facilitate attempts at replication
17	Procedural fidelity	Describe how procedural fidelity was evaluated in each phase
	ANALYSIS	
18	Analyses	Describe and justify all methods used to analyze data
RESULTS		
19	Sequence completed	For each participant, report the sequence actually completed, including the number of trials for each session for each case. For participant/s who did not complete, state when they stopped and the reasons
20	Outcomes and estimation	For each participant, report results, including raw data, for each target behavior and other outcome/s
21	Adverseevents	State whether or not any adverse events occurred for any participant and the phase in which they occurred
DISCUSSION		
22	Interpretation	Summarize findings and interpret the results in the context of current evidence
23	Limitations	Discuss limitations, addressing sources of potential bias and imprecision
24	Applicability	Discuss applicability and implications of the study findings
DOCUMENTATION		
25	Protocol	If available, state where a study protocol can be accessed
26	Funding	Identify source/s of funding and other support; describe the role of funders

Authors can use the checklist to help with writing a research report and readers (including journal editors/reviewers) can use the checklist to evaluate whether the report meets the points outlined in the guideline. Users will find the detailed SCRIBE 2016 E&E document (Tate et al., 2016) helpful for providing rationale for the items, with examples of adequate reporting from the literature.

## Postpublication activities

Following publication of this SCRIBE 2016 Statement and the E&E article (Tate et al., 2016), the next stage of activity focuses on further dissemination. Obtaining journal endorsement for the SCRIBE 2016 is a vital task because it has been demonstrated that journals that endorse specific reporting guidelines are associated with better reporting than journals where such endorsement does not exist (Turner et al., 2012). The SCRIBE project is indexed on the EQUATOR network (http://www.equator-network.org/) and a SCRIBE website (www.sydney.edu.au/medicine/research/scribe) provides information and links to the SCRIBE 2016 publications. SCRIBE users are encouraged to access the website and provide feedback on their experiences using the SCRIBE and suggestions for future revisions of the guideline. Future research will evaluate the uptake and impact of the SCRIBE 2016.

## Conclusions

We expect that the publication rate of single-case experiments and the research into single-case methodology will expand over the years, given the evidence of such a trend (e.g., Hammond & Gast, 2010) and also considering the recent interest shown in journal publication of special issues dedicated to single-case design research referred to earlier in this article. As is common for guidelines, the SCRIBE 2016 will likely require updates and revisions to remain current and aligned with the best evidence available on methodological standards.

We developed the SCRIBE 2016 to provide authors, journal reviewers, and editors with a recommended minimum set of items that should be addressed in reports describing single-case research. Adherence to the SCRIBE 2016 should improve the clarity, completeness, transparency, and accuracy of reporting single-case research in the behavioural sciences. In turn, this will facilitate (a) replication, which is of critical importance for establishing generality, (b) the coding of different aspects of the studies as potential moderators in meta-analysis, and (c) evaluation of the scientific quality of the research. All of these factors are relevant to the development of evidence-based practices.
